# Beta HPV Type 15 Can Interfere With NF-κB Activity and Apoptosis in Human Keratinocytes

**DOI:** 10.3389/fcimb.2020.00111

**Published:** 2020-03-18

**Authors:** Francesca Paolini, Marco Zaccarini, Arianna Francesconi, Luciano Mariani, Luca Muscardin, Pietro Donati, Aldo Venuti

**Affiliations:** ^1^HPV-Unit, IRCCS Regina Elena National Cancer Institute, Rome, Italy; ^2^UOSD Tumor Immunology and Immunotherapy, IRCCS Regina Elena National Cancer Institute, Rome, Italy; ^3^Laboratory of Cutaneous Histopathology, IRCCS San Gallicano Dermatologic Institute, Rome, Italy

**Keywords:** beta HPV, NF-κB, incontinentia pigmenti, IKK gamma, subungual tumor, apoptosis, RNA silencing, HPV15

## Abstract

E7 protein from cutaneous as well as mucosal HPV types can alter NF-κB activity. Conflicting literature data show a HPV-induced up- or down-regulation of the NF-κB pathway in different cell lines. In a previous study we detected the expression of E7 gene of HPV15 in a subungual tumor of a patient affected by incontinentia pigmenti (IP). IP is a rare X-linked genodermatosis in which the IKKγ gene is altered. From observations in transgenic IKKγ defective mice, it was suggested that IKK-deficient cells may undergo rapid hyper-proliferation and apoptosis/necrosis, leading to increased pro-inflammatory cytokine production in the neighboring IKK-positive cells. The objective of this study was to ascertain if beta HPV 15 can alter apoptosis and NF-κB pathway in normal and IKKγ-deficient keratinocytes. The human immortalized keratinocyte cell line (HaCaT), and human primary keratinocyte (HPK) cells were transduced with a retrovirus expressing E6–E7 proteins of HPV 15 and IKKγ was successful silenced mimicking the HPV15 infection and IP. HPV15 E6–E7 gene expression improved NF-κB activity in human keratinocytes even when IKKγ was silenced by siRNA. In IKKγ silenced keratinocyte cells, TNF-α-induced apoptosis was strongly reduced by the expression of HPV15 E6–E7 genes. Beta HPV15 exerted this anti-apoptotic activity by decreasing pro-apoptotic BAK and cleaved Caspase 3 proteins. In conclusion, we can speculate that presence of persistent infection by beta papillomavirus might influence the biological fate of IP by altering NF-κB activation and apoptosis in IKKγ mutated cells, favoring their survival and possibly the development of tumors in the late stage of disease. Taken together, our data reinforce the importance of host genetic background in the pathogenesis of HPV-associated skin lesions.

## Introduction

Human papillomaviruses (HPV) are double stranded DNA viruses which include types that infect human mucosa and those that are present in cutaneous sites. To date, more than 200 HPV types have been identified (Bzhalava et al., 2015), the majority of which belonging to the Alphapapillomavirus (α), Betapapillomavirus (β), and Gammapapillomavirus (γ) genera, depending on their epithelial tropism (de Villiers, 2013). The high risk Alphapapillomaviruses are linked to tumor development in several anatomic regions while Beta or Gamma papillomaviruses have been associated mostly with cutaneous tumors. Carcinogenesis mechanisms of Alpha vs. Beta/Gamma appear quite different with a more direct involvement of Alpha papillomavirus through the concerted activity of the E5, E6, and E7 oncogenes (Venuti et al., 2011). Beta papillomaviruses can exert their oncogenic activity when other factors are acting favoring cutaneous squamous cell carcinoma (SCC) development, i.e., genetic disorders as in epidermodysplasia verruciformis (EV), immunosuppression as in transplant patients, or UV induced DNA damage. Viral load analysis in SCC specimens of non-EV patients revealed that not all cancer cells contain a copy of the Beta HPV genome (Weissenborn et al., 2005). These data imply that Beta HPV types may act at an early stage of skin carcinogenesis and, after a full establishment of the cancer phenotype, viral DNA may not be required any longer and may be rapidly lost (Tommasino, 2017). However, in EV patients where mutations of EV genes (mainly EVER1 and EVER2 genes) alter the natural barrier to Beta-HPV DNA replication (Orth, 2008 review), high copy numbers of Beta HPV DNA can be found in lesions (Dell'Oste et al., 2009; Borgogna et al., 2012). Conflicting literature data showed an up- or a down-regulation of the NF-κB pathway in different cell lines expressing E6–E7 genes of different Beta HPVs. An increased activity was reported for HPV38 (Hussain et al., 2011) as well as for high risk alpha papillomavirus (Vandermark et al., 2012) whereas a reduced activity was showed for other Beta HPV (Byg et al., 2012).

The expression of E7 gene of HPV15 was reported in a subungual tumor of a patient affected by incontinentia pigmenti (IP) (Donati et al., 2009). IP is a rare X-linked genodermatosis in which the IKKγ gene is altered (Fusco et al., 2014). The disease evolves in four stages with appearance of subungual painful tumors in the late stage (STIP). From observations in transgenic IKKγ defective mice, it has been suggested that IKK-deficient cells may undergo rapid hyper-proliferation and necrosis, leading to increased pro-inflammatory cytokine production in the neighboring IKK-positive cells (Bodak et al., 2003).

The goal of this study was to ascertain if beta HPV 15 can alter apoptosis and NF-κB pathway in normal and IKKγ-deficient keratinocytes. Data from our experimentations showed that HPV 15 is able to interfere with apoptosis and NF-κB activity even in human keratinocytes where the IKKγ gene was silenced reproducing *in vitro* the IP genetic disorder. Consequences of this viral activity on the pathogenesis of STIP are discussed.

## Materials and Methods

### Cell Cultures

The spontaneously immortalized human keratinocyte cells (HaCaT) were grown in Dulbecco's modified Eagle's medium (DMEM) supplemented with 10% fetal bovine serum (FBS), glutamine, and penicillin-streptomycin (Gibco, Karlsruhe, Germany) to 70–80% confluence prior passages.

Phoenix A helper-virus free ecotropic packaging cells were utilized. This cell line is easy to transform by standard transfection protocols and allows to assembly infectious progeny in a short time. Phoenix A cells were maintained in High Glucose DMEM medium supplemented with 10% FBS. Human primary keratinocytes (HPK) (kindly provided by Giorgia Cardinali and Daniela Kovacs S.) were maintained in KGM-Gold™ Keratinocyte Growth Medium (Lonza Basel, Switzerland) in monolayer culture. HPK at second/third passage were used for experimental procedures.

### HPV 15 E6–E7 Retrovirus Preparation

All the procedures involving retrovirus utilization were performed in P2 laboratories. Phoenix A cells were seeded at 10^4^ cell/cm^2^ and after 24 h were incubated for 30 min at 37°C with complete DMEM containing 25 μM Cloroquine diphosphate. After incubation, a DNA Calcium Phosphate mixture containing HPV 15 E6–E7 recombinant pLXSN Retroviral Vector (a generous gift of M Feltkamp), 0.25 M of CaCl_2_ and 50 mM of N,N-bis (2- hydroxyl ethyl)- 2-aminoethansulfonic acid was added dropwise to the Phoenix A cells under gentle agitation for 18 h. Then, the cells were washed with PBS and were incubated with complete DMEM at 32°C with 5% CO_2_ for 48 h. The medium containing HPV 15 E6–E7 bearing retroviral progenies was clarified by centrifugation, aliquoted and stored at −80°C for later use.

### Silencing of IKKγ by siRNA

In order to achieve 30% confluence at the time of transfection, 1 day before, the cells were seeded without antibiotics. IKKγ siRNA was synthesized *in vitro* by Silencer™ siRNA Construction Kit (Thermo Fischer, Milan, IT) with the following oligomers:

Sense 5′-AAAAGATTGTGATGGAGACCGCCTGTCTC-3′;

Antisense 5′-AACGGTCTCCATCACAATCTTCCTGTCTC-3′.

siRNA and Lipofectamine™ 2000 (Invitrogen, Milan, Italy) were diluted in Opti-MEM I (Gibco, Milan, Italy) without serum according manufacturer instruction. Cells were transfected with 100 nM of IKKγ siRNA and were incubated at 37°C in a CO_2_ incubator. Western blot analysis at 24-48-72 h showed almost no IKKγ protein in silenced cells; only a faint band can be barely detected, indicating presence of few un-transfected cells (Supplementary Figure 1A). All the experiments were performed at 48 h and silencing efficiency was evaluated in w/b as showed in Supplementary Figures 1A,B.

### Infection by Recombinant Retrovirus Expressing HPV15 E6–E7

Twenty-four hour before infection, HaCaT or HPK cells were seeded at 5 × 10^4^ cell/cm^2^. The infection mixture containing HPV15 E6–E7 retrovirus and Polybrene (5 μg/ml) was added to the cells. Flasks were then centrifuged for 30 min at cold temperature and incubated for 18 h at 32°C in a 5% CO_2_ atmosphere. Then, infected cells were maintained in 37°C, 5% CO_2_ conditions and medium was changed after 24 h. HaCaT cells were subjected to antibiotic selection (400 μg/ml of G418 - Gibco Life Technologies). Typically, 90–100% of the HaCaT cells were infected by recombinant retrovirus and single clones isolated and sub-cultivated in presence of G418. The presence of the specific E6 and E7 mRNA was confirmed by reverse transcriptase (RT)-PCR. HPK cells did not undertake selection and were utilized shortly after transduction.

### RT-PCR

Cell RNA preparation was performed using the RNeasy Plus Mini kit (QIAGEN, Milan, Italy), according to the manufacturer's instructions. Total RNA was DNase treated, retro-transcribed into cDNAs, as described by the manufacturer (GeneAmp RNA PCR kit Applied Biosystem, Foster City, CA), and analyzed by PCR with Platinum TaqDNA polymerase (Invitrogen, Milan, Italy) using specific primers for HPV 15 E6 and E7 genes as previously described (Dang et al., 2006; Donati et al., 2009). Control of contamination with genomic DNA was a mock reverse transcription containing all the RT-PCR reagents, except the reverse transcriptase. The amplified products were resolved in sybr green stained agarose gels.

### TNF-α Stimulation

Cells were incubated with TNF-α (10 ng/ml) for the time specified for each experiment. The TNF-α dosage was chosen according to reported data (Hussain et al., 2011). The stimulation was terminated by replacing the medium with cold PBS, followed by cell lyse for Luciferase assays or cell death ELISA and by protein harvesting for western blot assay.

### p16^INK4a^ Detection

The p16^INK4a^ detection was carried out by a commercial kit (histologic CINtec Plus, Roche, Milan, Italy) on 5 μm sections from formalin fixed paraffin embedded blocks of clinical sample (Paolini et al., 2011). In cell lysates, presence of p16^INK4a^ was ascertained by western blot. Lysates of HeLa cells expressing E6 and E7 of HPV 18 were utilized as positive control.

### NF-κB Activity by Luciferase Assays

Cells were seeded in 24-well plates at 5 × 10^4^ cells per well. Twenty-four h. after IKKγ silencing, the cells were transfected by Lipofectamine™ 2000 with a plasmid carrying an NF-κB dependent Firefly luciferase reporter construct with three NF-κB binding sites (κB) upstream of the luciferase gene (κB)3-Luc (1 μg) (BCCM^TM^/LMBP, Gent, Belgium), together with a Renilla luciferase expressing control plasmid (1 ng). Twenty-four hours after transfection the cells were incubated with TNF-α for 6 h. The activity of NF-κB was measured in a GloMax® 20/20 Luminometer (Promega, Fitchburg, WI) using the luciferase assay system (Promega, Fitchburg, Wisconsin). Results were normalized to Renilla luciferase light emission.

### Immunoblotting

For western blot analysis, total sample lysates were separated by a 12% SDS-PAGE and transferred onto an Immune-Blot^TM^ polyvinylidene difluoride (PVDF) membrane (Bio-Rad Laboratories, CA, USA). After blocking in 5% milk (Bio-Rad Laboratories, CA, USA) the membrane was probed overnight at 4°C. with anti-IKK-γ antibody (ab178872), anti-NF-κB p65, anti-CDKN2A/p16^INK4a^ (ab108349), anti-BAK antibody (Y164) (ab32371), anti-cleaved Caspase 3 (Asp175), or β-actin (ab8227). Horse radish peroxidase (HRP) conjugated goat anti mouse IgG antibodies and goat anti rabbit IgG antibodies were utilized as secondary antibodies (1 h at room temperature). All antibodies were from Abcam (Cambridge, MA) except anti-cleaved Caspase 3 that was from Cell Signaling Technology (Beverly, MA). After washings, membranes were incubated in ECL^TM^ Western blotting detection reagent (GE Healthcare, Buckinghamshire, UK) and analyzed by exposure to x-ray films or by direct acquisition on ChemiDoc Imaging System (BioRad, Milan, Italy) for the other proteins.

### Apoptosis

HaCaT or HPK seeding and experimental conditions were as for luciferase assay. Briefly, cells were lysed and apoptosis measured by a commercial kit revealing presence of apoptotic cytoplasmic histone-associated DNA fragments (Cell Death Detection ELISA, SigmaAldrich, Milan, Italy).

### Cytokine and Chemokine Assay

HaCaT and HaCaT 15 E6–E7 cells were seeded at the same density (8 × 10^4^ cells/cm^2^). Cells were TNF-α or mock-treated for 6 h and supernatants collected after 24 h. Supernatants were centrifuged for 5 min at 1,200 × g to eliminate cell debris and thereafter diluted for Luminex assay. Cytokines and chemokines were analyzed using a Bio-Plex human chemokine 40-plex assay panel (Bio-Rad Laboratories Inc. Hercules, CA, U.S.A.) according to manufacturer's instructions. Briefly, capture antibody-coupled beads were allowed to react with triplicated samples of the diluted supernatants. After performing a series of washes to remove unbound materials, a biotinylated detection antibody specific for a different epitope on the target was added to the beads. The reaction mixture was detected by streptavidin-phycoerythrin, which binds sandwich complexes via the biotinylated detection antibody. The cytokine/chemokine contents of each well were identified and quantified against standard samples using Bio-Plex Magpix apparatus (Bio-Rad Laboratories Inc. Hercules, CA, U.S.A.).

## Results

### HPV 15 and p16^INK4a^ Expression

**Figure 1:**
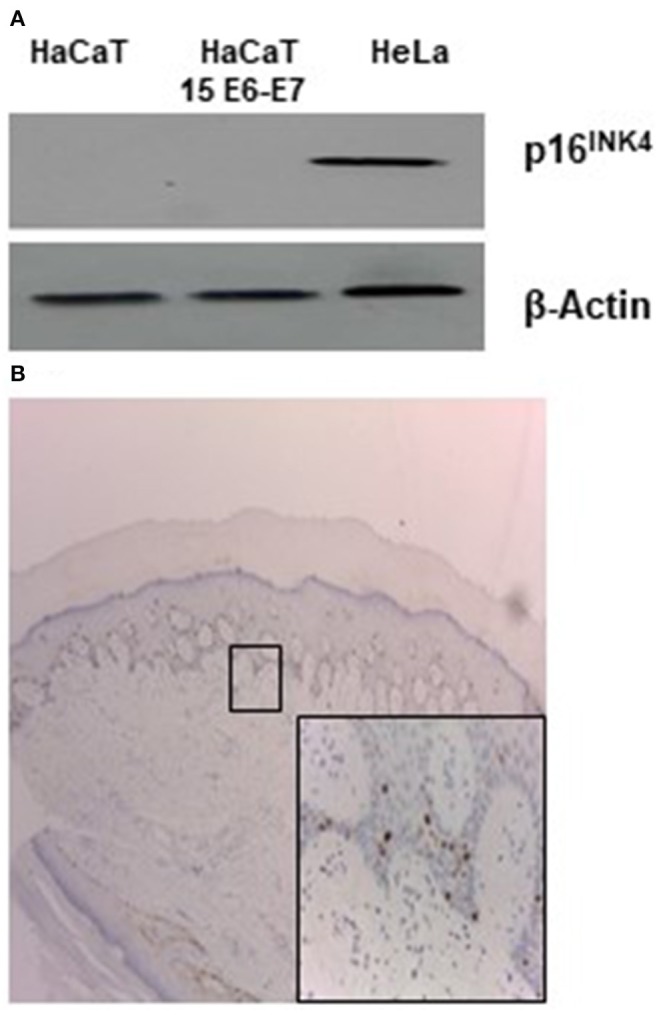
HPV15 and p16^INK4a^ stain. **(A)** Western blot analysis for p16^INK4a^ in HaCaT and HaCaT 15 E6-E7. Hela cell lysate was utilized as positive control. **(B)** Immunohistochemistry for p16^INK4a^ of clinical sample from STIP patient (Donati et al., 2009) (magnification 100X). In the inset, rare positive cells referable to melanocytes (magnification 200X).

Few literature data exist regarding the specific biological/molecular activity of HPV 15. It was reported that HPV 15 can alter morphology of keratinocytes organotypic culture and it was found in basal cell carcinoma (BCC) in association with p16INK4a positivity. For alpha HPV, it is well-known that the continuous high expression of E6 and E7 oncogenes can lead to overexpression of p16^INK4a^ protein. To ascertain if this activity was retained in our experimental conditions p16^INK4a^ expression was analyzed in HaCaT 15 E6-E7. No p16^INK4a^ protein was detected by western blot (Figure 1A). Keratinocytes are cells subjected to differentiation and p16^INK4a^ expression should be measured in differentiating/physiological conditions. Since original biopsy of the patient with STIP (Donati et al., 2009) was still available in paraffin block, p16^INK4a^ stain was evaluated and no positive signal was detected in epithelial cells, confirming the absence of p16^INK4a^ overexpression in HPV15 positive cells (Figure 1B).

### HPV15 Increases NF-κB Activation by TNF-α

**Figure 2:**
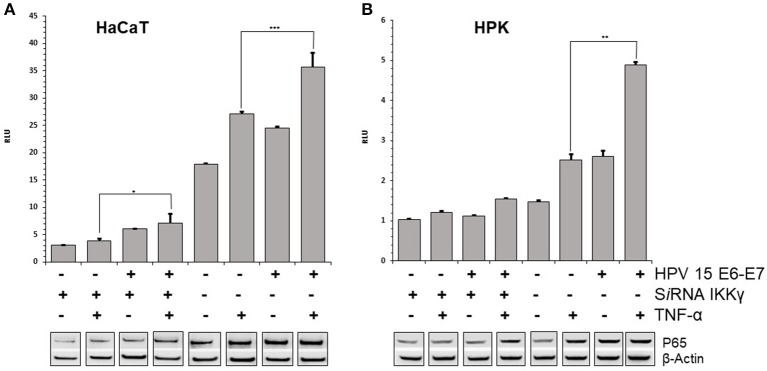
HPV15 affects TNF-α-induced NF-κB activity. HaCaT **(A)** and HPK **(B)** cells were utlized. HPV 15 transduction, IKKγ gene silencing and TNF-α treatment (10 ng/ml) for 6 h are indicated. NF-κB activity was measured by a GloMax® 20/20 Luminometer using a luciferase assay system. Data are mean of triplicate samples and are expressed in relative light unit (RLU) as ratio between Firefly and Renilla luciferase activity. Error bars = ±SD. *p < 0.05; **p < 0.001; ***p < 0.0001 by unpaired *t*-test. p65 subunit of NF-κB complex was detected by Western blot with anti-NF-κB p65 as described in section Materials and Methods. All these experiments were repeated two times with similar results.

E7 gene of Beta HPV can affect the TNFα-induced activity of NF-κB complex. The p65 subunit of NF-κB transcription complex was slightly increased after TNF-α stimulation in HPV15 E6–E7 expressing- vs. normal-HaCaT cells (Figure 2A). Similar results were obtained by p65 immunoblotting with HPV15 E6–E7 transduced HPK cells (Figure 2B). On the other hand, to have a picture on the functional activity of NF-κB in presence of HPV15 E6-E7 genes, a luciferase assay was conducted with a plasmid carrying Firefly luciferase gene under the control of three NF-κB responding elements. HaCaT and HaCaT 15 E6-E7 cells were transfected with luciferase expressing plasmids and thereafter treated with or without TNF-α for 6 h. Cells were harvested, and luciferase activity measured in a luminometer. In Figure 2, data from HaCaT and HaCaT 15 E6–E7 with or without IKKγ silencing were presented. As clearly showed after normalization by Renilla luciferase activity, the presence of E6–E7 genes improved the NF-κB activity even in the absence of IKKγ (silenced) whose lack greatly reduced the activity of the NF-κB complex (Figure 2). Indeed, normal-HaCaT cells showed a dramatically reduced NF-κB activity upon TNF-α stimulation when IKKγ was silenced. The increased NF-κB activity was highly statistically significant after TNF-α treatment in HaCaT 15 E6–E7 vs. control cells (no HPV). These results were substantially confirmed in TNF-α or mock treated HPK for 6 h. Indeed, HPK 15 E6–E7 showed an increased NF-κB activity even in the absence of IKKγ (Figure 2). The highest statistically significant differences were recorded in HPK 15 E6–E7 vs. control cells (no HPV).

### HPV15 Affects TNF-α-Induced Apoptosis

**Figure 3:**
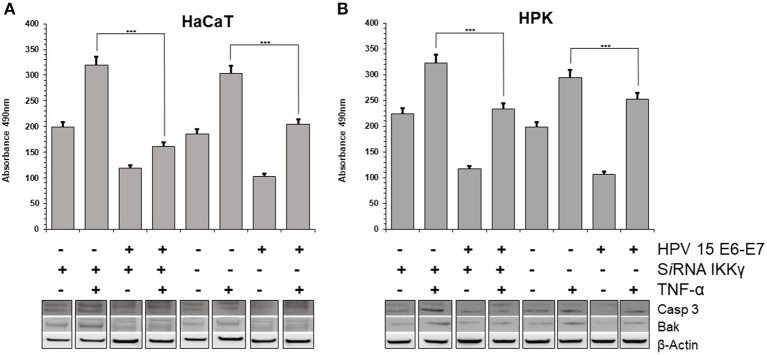
HPV15 affects TNF-α-induced apoptosis. HaCaT **(A)** and HPK **(B)** cells were utlized. HPV 15 transduction, IKKγ gene silencing and TNF-α treatment (10 ng/ml) for 6 h are indicated. Apoptosis was measured by a Cell Death ELISA test according to manufacturer's instruction in triplicate samples. Error bars = ±SD. ***p < 0.0001 by unpaired *t*-test. Activation of apoptotic pathway was analyzed in cell lysates by Western blots with anti-cleaved Caspase 3 or anti-BAK antibodies, as described in section Materials and Methods. All these experiments were repeated twice with similar results.

Stress response to different chemical or physical inductors can lead to cell apoptosis. It was suggested that TNF-α prepares keratinocytes to respond quickly to additional signals: if these promote apoptosis, the cells will die; if they promote survival, apoptosis will be inhibited (Banno et al., 2004). In the literature was reported that TNF-α induces growth arrest in keratinocytes, possibly through differentiation (Basile et al., 2001). However, it was also reported that HPV 38 is able to counteract the apoptosis induced by TNF-α in human keratinocytes (Hussain et al., 2011). To assess the possible apoptotic response induced by TNF-α, the cleavage of the caspase-3, which is a critical initial event closely associated with apoptosis, was analyzed HaCaT and HPK cells. In both cell lines, particularly in IKKγ silenced cells, the cleaved forms of Caspase 3 were increased, indicating activation of this apoptotic pathway by TNF-α. On the contrary, in HPV15 E6-E7 expressing cells the cleaved forms of Caspase 3 were dramatically reduced (Figure 3). In addition, apoptosis is regulated by Bcl-2 family proteins, including pro-apoptotic protein such as Bak (Hardwick and Soane, 2013). Interestingly, HPVs are able to suppress apoptosis by degrading Bak (Underbrink et al., 2008). In both cell lines, HPV15 E6-E7 expressing cells showed decrease levels of Bak expression vs. control cells (no HPV), indicating a possible interaction of HPV15 with this cellular pathway, as already reported for other Beta HPV (Underbrink et al., 2008). Caspase-3 and Bak data indicated that apoptosis was occurring in our cell models and HPV 15 was able to counteract it, therefore late apoptosis was measured to confirm these results. Results from cell dead ELISA test showed that TNF-α-induced apoptosis was reduced in HaCaT 15 E6–E7 and HPK 15 E6–E7 cells (Figure 3). Indeed, TNF-α exposure, as expected from Caspase 3 and Bak data, did increase the level of apoptosis in control cells after IKKγ silencing, whereas presence of HPV15 E6–E7 was able to reduce this apoptosis (Figure 3). The apoptosis reduction by HPV 15 was statistically significant.

### HPV15 Alters Chemo/Cytokine Asset

**Figure 4:**
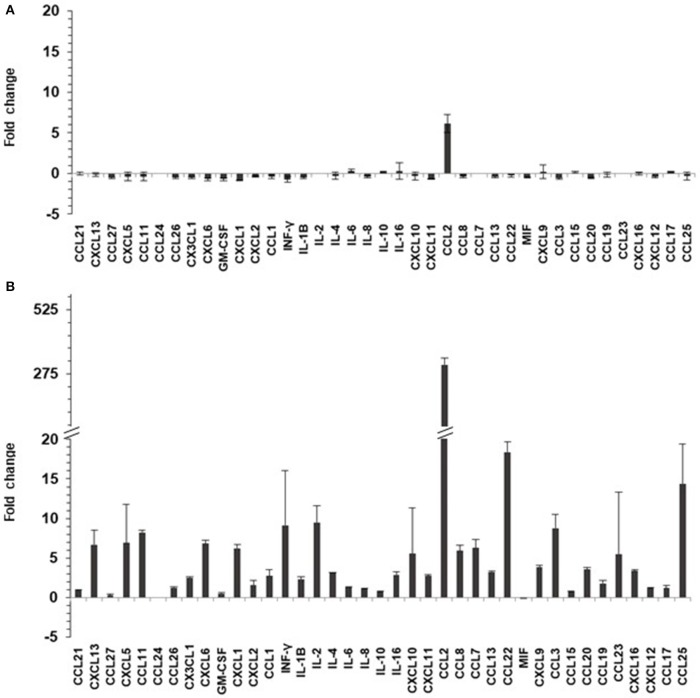
HPV 15 alters cyto/chemokine production profile. The amount of cyto/chemokines was measured as pg/ml by Multiplex system technology in the supernatants of HaCaT cells plated at 8 × 10^4^ cells/cm^2^, as in section Materials and Methods. Data are expressed as fold change of HaCaT 15 E6–E7 vs. HaCaT control and are means ± SD of two experiments. **(A)** No treatment; **(B)** TNF-α treatment (10 ng/ml) for 6 h.

TNF-α can induce secretion of chemo/cytokines in keratinocytes via NF-κB pathway. Twenty-four hours after TNF-α or mock-treatments (for 6 h), culture supernatants of HaCaT and HaCaT 15 E6–E7 cells were collected and analyzed for the presence of 40 different cyto/chemokines (BioPlex assay). Chemokine and cytokine quantity levels were altered by the presence of HPV15 with a strong increase after TNF-α stimulation. In particular, the presence of HPV15 E6–E7 produced an impressive increase (>300 fold increase) of CCL2 production after TNF-α stimulation, as showed in Figure 4.

## Discussion

*In vitro* and *in vivo* experimental models of Beta papillomavirus biology have underlined the properties of E6 and E7 from several Beta HPV types in deregulating fundamental cellular events intimately linked to transformation, such as cell cycle progression, apoptosis, differentiation, and DNA repair (Howley and Pfister, 2015; Tommasino, 2017). Beta papillomaviruses show different activity in cell transformation and interaction with cellular pathways. In previous works, we detected HPV15 mRNA in a subungual tumor of IP patient as well as HPV15 DNA in basal cell carcinoma (BCC) in association with p16^INK4a^ positivity (Donati et al., 2009; Paolini et al., 2011). This last observation may suggest a possible alteration of pRB pathway as for High-Risk Alpha papillomavirus. Our data seem to exclude this hypothesis because we did not detect any increased expression of p16^INK4a^ protein in keratinocytes transfected with E6–E7 genes of HPV15. In addition, p16^INK4a^ stain of HPV15 positive subungual tumor failed to detect positive stains. This result may indicate that high expression of p16^INK4a^ in BCC was linked to other tumor-induced alterations rather than to expression of HPV15 E7 gene. However, Boxman et al. (2001) showed an altered morphology of organotypic culture of HPV15 transfected cells, suggesting that HPV 15 may alter keratinocyte differentiation.

Thus, the biological activity of this HPV 15 was further analyzed in an *in vitro* experimental model resembling the IP disease. HaCaT and HPK cells silenced for IKKγ gene were unable to produce the corresponding protein and the NF-κB response to the TNF-α stimulus was dramatically reduced, suggesting that these keratinocytes are similar to that of IP patient where X chromosome carrying IKKγ mutation is active. In these cells, IKKγ silencing was also associated with an increased apoptosis that is a key feature of IP pathology (Makris et al., 2000). Both NF-κB and apoptosis were altered by the presence of E6 and E7 genes of HPV 15. NF-κB activity increased by E6–E7 even in the presence of silenced IKKγ gene. Conflicting data were reported about E7 activity of Beta HPV on NF-κB activity. Byg et al. (2012) described a reduced activity whereas Hussain et al. (2011) showed an increased activity as reported also for High-Risk Alpha papillomavirus (Vandermark et al., 2012). The Hussain's study was performed with HPV 38 that is a Beta-2 type such as HPV 15. Our findings are in accordance with the HPV 38 results. However, in Byg's study other Beta-2 types were utilized. These puzzling results can be explained by the different cell lines utilized by Byg et al., U2OS instead of keratinocytes. It is conceivable that the interaction of Beta-papillomavirus with NF-κB activity is depending on cell type and/or on specific type of HPV, as showed for interactions with other cell pathways (Wendel and Wallace, 2017, review). To clarify this point, NK-κB activity was measured in human primary keratinocytes, the physiological target cells of HPV. Experimental results showed that HPV15 was able to increase the NK-κB activity in response to TNF-α stimulation, confirming and strengthening the results obtained with HaCaT cells. Thus, HPV15 E6–E7 seems to activate NF-κB even when IKKγ is silenced allowing to speculate about a possible direct interaction between E6 or E7 with NF-κB complex proteins. However, the lack of suitable antibodies for HPV15 E6 or E7 proteins hampered to elucidate this point.

In both cell lines, HaCaT and HPK, an inhibition of apoptosis in IKKγ silenced as well as normal cells was clearly detected, although in HaCaT cells p53 is mutated but cells can undergo apoptosis using alternative pathways. In addition, we showed that Bak pathway was activated and HPV 15 E6–E7 was able to decrease its activation. E6 from Beta HPV types promotes the degradation of the pro-apoptotic protein Bak, a member of the Bcl-2 family (Jackson et al., 2000; Underbrink et al., 2008). In chemical or UV radiation stressed cells, Bak is post-translationally modified and acquires the properties to oligomerize at the mitochondrial membrane, promoting formation of pores, release of cytochrome C from the mitochondria, induction of caspases, and activation of the apoptotic cascades (Dewson et al., 2009). E6 of Beta-1 HPV5 and HPV8 directly interacts with Bak, inducing its degradation through the proteasome pathway, thus preventing the induced apoptotic response (Underbrink et al., 2008; Holloway et al., 2015). In HaCaT and HPK, the anti-apoptotic activity of HPV15 E6–E7 was also confirmed by measuring the level of cleaved Caspase 3. Indeed, Caspase-3 is a key point of apoptosis and its activation requires proteolytic processing of its inactive form into activated p17 and p12 fragments. These fragments were increased in HaCaT and HPK upon TNF-α stimulation indicating the activation of this pro-apoptotic pathway. The expression of HPV 15 E6–E7 dramatically reduced this activation even in IKKγ silenced keratinocytes suggesting that HPV15 is able to affect apoptosis in human damaged keratinocytes as those of IP patients. In IP, the IKKγ mutate gene encodes for a non-functioning protein, incapable to activate the NF-κB pathway, therefore, cells in which the mutant X chromosome remains active are devoid of NF-κB activation and become more sensitive to apoptosis and hyperproliferation. Observations in transgenic mice led to the development of a pathophysiological model in which IKK deficient cells can undergo rapid hyperproliferation and necrosis, leading to increased proinflammatory cytokine production in the neighboring normal cells (Bodak et al., 2003). In fact, most of the cytokines whose the expression was high in the skin of the transgenic mouse are under NF-κB control and cannot be produced by IKK deficient cells. An effect of the proinflammatory cytokines might be the enhancement of apoptosis in the mutated cells. Actually, numerous apoptotic cells were identified by histologic examination of skin biopsy specimens of IP patients and TUNEL assays in transgenic mice (Makris et al., 2000). Thus, the cells where the mutant X chromosome is active are then gradually eliminated through apoptosis, leading to the classic progressive resolution of the skin lesions. It was suggested that the persistence of some mutated cells could explain the documented reactivation of the inflammatory stage with hyper proliferation, inflammation, and apoptosis (Makris et al., 2000). The role of pro-inflammatory cytokines such as TNF-α was postulated, enhancing hyper-proliferation, necrosis, and apoptosis in the persistent IKKγ-deficient cells, abnormally sensitive to TNFα-induced apoptosis (Beg and Baltimore, 1996; van Antwerp et al., 1996; Seitz et al., 2000). Data from our experiments indicated that HPV15 E6–E7 genes alter cyto/chemokine asset producing their impressive increase, in particular upon TNFα stimulation. These cyto-/chemokines are produced by the activation of NF-κB pathway that is up-regulated by HPV15 E6/E7. Among them it is interesting to note that CCL2 was highly stimulated by the presence of HPV15 E6–E7 upon TNF-α treatment. This chemokine could favor *in vivo* the induction of a pro-tumorigenic and immunosuppressive microenvironment. The persistence of viral infection together with an inflammatory background is a well-recognized factor of HPV induced hyper-proliferation. The rare and late appearance of painful subungual tumors in IP patients is consistent with the long latency of HPV infection. In addition, subungual localization can be considered a hot spot of transformation by High Risk HPV (Venuti et al., 1994; Grundmeier et al., 2011).

We were unable to analyze other tumors from IP patients and two single reports failed to detect HPV in STIP (Simmons et al., 1986; Kibbi et al., 2018). However, in that reports the authors attempted to identify HPV virus by immunostaining that is less sensitive than PCR assay utilized in our study. In conclusion, we can speculate that presence of persistent HPV infection might influence the biological fate of IP by altering NF-κB activation and apoptosis in IKKγ mutated cells, favoring their survival and possibly the development of subungual tumors in the late stage of disease. A screening for HPV could be helpful in assessing cancer risk in patients with IP or other genetic disorders. Further studies are needed to support the possibility of such screening subjected to the identification of possible high risk Beta-HPVs (Bouvard et al., 2009).

## Data Availability Statement

The datasets generated for this study are available on request to the corresponding author and part of them are in the GARRbox repository.

## Author Contributions

PD and AV conceived and designed the experiments. FP, MZ, and AF performed the experiments. LMa and LMu analyzed the data. FP and AV wrote the manuscript. All authors read and approved the final manuscript.

### Conflict of Interest

The authors declare that the research was conducted in the absence of any commercial or financial relationships that could be construed as a potential conflict of interest.

## Supplementary Material

Supplementary Figure 1 IKKγ silencing. HaCaT and HPK cell lines expressing HPV15 E6–E7 genes were obtained by recombinant retrovirus and Western blot analysis for IKKγ protein performed as in Methods. **(A)** Western blot analysis for IKKγ protein at different time interval after silencing in HaCaT 15 E6–E7. Hela cell lysate was utilized as positive control. **(B)** Western blot for IKKγ protein in HaCaT and HPK cell lysates from different experimental conditions. HPV 15 transduction, IKKγ gene silencing and TNF-α treatment (10 ng/ml) for 6 h were indicated.
