# Supplementary figures and images for: Beta HPV Type 15 Can Interfere With NF-κB Activity and Apoptosis in Human Keratinocytes
Source: Front Cell Infect Microbiol. 2020 Mar 18;10:111. doi: 10.3389/fcimb.2020.00111 (PMC7093591; doi:10.3389/fcimb.2020.00111)

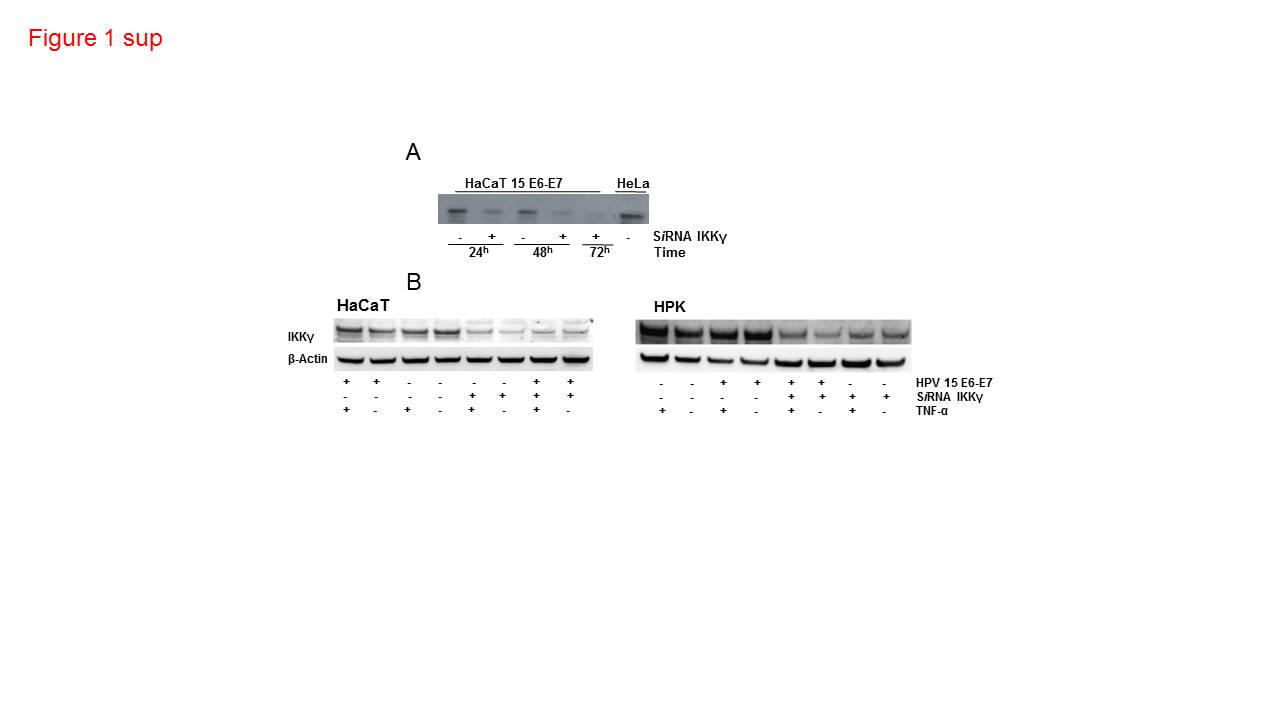

Supplement: Supplementary Figure 1 — IKKγ silencing. HaCaT and HPK cell lines expressing HPV15 E6–E7 genes were obtained by recombinant retrovirus and Western blot analysis for IKKγ protein performed as in Methods. (A) Western blot analysis for IKKγ protein at different time interval after silencing in HaCaT 15 E6–E7. Hela cell lysate was utilized as positive control. (B) Western blot for IKKγ protein in HaCaT and HPK cell lysates from different experimental conditions. HPV 15 transduction, IKKγ gene silencing and TNF-α treatment (10 ng/ml) for 6 h were indicated. [file Image_1.JPEG]
